# North American Urban Indigenous Food Systems

**DOI:** 10.1016/j.cdnut.2025.104542

**Published:** 2025-01-06

**Authors:** Christine Bruno, Clifton Bruno, Alexandria Cruz, Sara Moncada, Kaitlyn Patterson, Brooke Rodriguez, Dave Skene, Hannah Neufeld

**Affiliations:** 1 Indigenous Consultants, Portland, OR, United States; 2 Grinding Stone Collective, Glendale, NY, United States; 3 The Culture Conservancy, San Francisco Bay Area, CA, United States; 4 All Our Relations Land Trust, Katarokwi, Ontario, Canada; 5 School of Public Health Sciences, The University of Waterloo, Ontario, Canada; 6 The Wisahkotewinowak Collective, Kitchener, Ontario, Canada

**Keywords:** Indigenous food systems, urban, food sovereignty, food environments, Indigenous health

## Abstract

These compiled stories were shared as part of a moderated panel during the Sixth Annual Conference on Native American Nutrition that was held in September 2023 at Mystic Lake Center on the lands of the Shakopee Mdewakanton Sioux Community within present day Prior Lake, Minnesota. Other members of the North American Urban Indigenous Food System network supported by the University of Waterloo and the Food and Agriculture Organization of the United Nations were also invited to contribute their perspectives on the inspiring community-led work that is taking place within their respective urban spaces and places across Turtle Island. Network members shared the unique circumstances associated with finding spaces and places of connection and belonging in the city and how practices of Indigenous food sovereignty through knowledge sharing and ecological responsibilities provide healing through relationship.

## Introduction

During the Sixth Annual Conference on Native American Nutrition, a panel on the topic of North American urban Indigenous food systems took place that included the experiences and stories of the inspiring work being led within cities across Turtle Island. The participants on the panel included members of a North American Urban Indigenous Food Systems network supported by the University of Waterloo and the Food and Agriculture Organization (FAO) of the United Nations Liaison Office for North America. The network represented by panel members was initiated after several conversations between FAO and researchers engaged in this area with the knowledge that a significant and growing number of Indigenous Peoples’ communities live in cities, both because of historical displacements and more recent trends. Approximately 71% of American Indians and Alaska Natives [1] and close to half of Indigenous Peoples within Canada live in urban centers [2]. The forces of a cash economy along with ecosystem and cultural dispossession and destruction have influenced a movement toward urbanization, with global demographic trends indicating that Indigenous communities are becoming more urban [3]. Indigenous Peoples increasingly identify cities as their home communities as they establish cultural spaces and meaningful connections with the lands and waters of these new and emerging environments.

Urban Indigenous communities across Turtle Island are interfacing with complex and changing food environments owing to persisting colonial beliefs that cities are settler spaces [4,5]. Urban communities have encountered a loss of control over the foods they interact with and often struggle to access Indigenous foods after moving to urban areas [6]. On an international scale, 1.7 billion of the world’s 2.2 billion people experiencing moderate or severe food insecurity live in urban centers [7]. The ability of urban communities to realize their right to food is therefore a critical challenge, particularly in the context of climate change, political instability, increased inequality, internal conflicts, and rapidly increasing urban populations. Despite these challenges, many Indigenous communities are demonstrating innovative and emerging pathways toward revitalizing and redefining Indigenous foodways and self-determination through food skills workshops and land-based programming [8,9]. These practices collectively aim toward Indigenous food sovereignty (IFS), support food security, dietary health and wellbeing, and also emphasize the importance of responsibilities for sustainably caring for land and community amidst growing social and climate change pressures in cities.

Since June 2022, a growing circle of individuals representing urban Indigenous communities, collectives and organizations have been meeting to strengthen a North American network of knowledge pathways, using food as a starting point for action, to promote conversations and opportunities. Representatives of 3 Indigenous groups involved in the network presented as part of a panel in the fall of 2023 at the Native American Nutrition Conference. Stories were shared from Dave Skene (The Wisahkotewinowak Collective), Christine and Clifton Bruno (Indigenous educators and consultants), and Sara Moncada (The Culture Conservancy). Three additional representatives in the network have shared their experiences within this paper including founders of the Grinding Stone Collective (GSC) (Alexandria Cruz and Brooke Rodriguez) along with All Our Relations Land Trust (Kaitlyn Patterson). These 5 commentaries represent the range of synergies that have developed as part of this growing network and highlight the spaces and places they participate in to actively maintain and strengthen their relationships with land and food through IFS.

All contributing authors have created and reviewed these written pieces that begin with introductions connecting them to their ancestors and go on to describe the environments they work within along with the communities they support. Their sharing across Turtle Island begins from East to West.

*Hannah Neufeld, School of Public Health Sciences, The University of Waterloo, Ontario, Canada; Mikaila Way, Indigenous Peoples’ Liaison for North America, Food and Agriculture Organization of the United Nations.*

## Alexandria Cruz and Brooke Rodriguez (Grinding Stone Collective, Glendale, New York, United States)

I am Alexandria Cruz (Borikua Taino | Yucayeke Abacoa) and live in occupied Lenape/Canarsie territory now known as Brooklyn, New York. I work as the Program and Communications Manager at GSC Inc. and Board Secretary at the Borikua Taino Foundation. I am a writer and a scholarship graduate from Bard College via their program at the Brooklyn Public Library. I previously graduated from the New York City (NYC) Museum School and completed the Food and Sustainability Certificate Program at the European Institute of Innovation for Sustainability along with the Dialects of Decolonization activist program through the Center for Embodied Pedagogy and Action with a focus on Puerto Rico. I have worked as Program Coordinator for University Open Air, a program dedicated to employing immigrant professors, and at various museums throughout NYC.

My name is Brooke Rodriguez (BorikuaTaino | Yucayeke Abacoa) and I am a dedicated mother living in Munsee Lenape territory. I am the founder of GSC, co-owner of Buffalo Jump NYC, and Board President of the Borikua Taino Foundation. As GSC’s executive director, I lead initiatives uplifting Indigenous communities through events, workshops, and the First Foods Program. I am committed to preserving Borikua Taíno’s cultural identity as a founding member of the Borikua Taino Foundation and a driving force for self-determination. Previously I supported the Indigenous Medicine Collective and in 2022, I received a fellowship in Solve MIT’s Indigenous Communities cohort. In total, I have over 20 years of advocacy and academic roots in agriculture, animal husbandry, biology, and anthropology.

GSC is a grassroots nonprofit with a core mission to build strong, self-sufficient communities and connections between urban and rural Indigenous Peoples. We are committed to creating innovative, impactful, and sustainable solutions to the challenges facing us as Indigenous People today. We accomplish this by working with both Technology and Traditional Ecological Knowledge (TEK) to promote Indigenous cultures and knowledges through education, collaboration, and innovation. We strive to create new opportunities for Indigenous People to share their stories, values, and history with the larger intertribal community and allies. Through our initiatives, we unite Indigenous People from all parts of Turtle Island and foster empowered intertribal connections. Our First Foods Program was founded in 2020 and addresses the dual challenges of reconnecting urban Indigenous communities with their food cultures while combating food insecurity. First Foods champions food sovereignty through a system rooted in Education, Reciprocity, and Action and empowers communities to govern their food systems from using both Ancestral and Contemporary Knowledges. Our educational initiatives include free classes, food heritage events, community discussions, and cooking/gardening demonstrations where we aim to reduce access barriers. By preserving and sharing Indigenous Knowledges, our program fosters IFS, promotes biodiversity stewardship, alternative food preparation methods, and healthy living outside of industrial food systems.

GSC has worked with Indigenous Peoples from across Turtle Island and Abya Yala. These urban settings create a condition where many Indigenous Peoples feel disconnected from their roots or tokenized by their position within the broader movement to diversify work and educational settings. Throughout our experiences there is a profound joy that has moved teachers, participants, and even us to tears upon sharing foods from their homelands. Our events serve as a bridge to traditions for the restoration of our ancestral connections. Indigenous foods in these contexts, awaken memories rooted in the cultural significance of communal eating that is so central to Indigenous foodways. It is not just the food itself but the act of sharing the significance of our unique culinary traditions. Food is more than nourishment. It is a love letter from our ancestors who first cultivated and harvested these foods through precolonial scientific methods. Indigenous Knowledge Systems (IKS) and TEK represent centuries old observations, experimentations, and adaptations passed down orally [10]. Our ancestors had to test which foods were safe, learn the conditions they needed to thrive in, and how best to prepare them for consumption. This required time, knowledge, and skills that have been passed down through the reclamation of these ancestral gifts. IKS and TEK do not exist in tribal silos. We have always shared our findings across territories to advance our foodways. Even with the colonial disruptions, North American Indigenous foods have become part of staple dishes miles from where they originated [11]. They, like we, have had to evolve to new and ever-changing conditions and environments. In the wake of climate change, our gifts and knowledges are being called upon once more as people return to communal sowing, harvest, and preservation. We feel pride in sharing our grandparents’ recipes and teaching the next generation of land stewards what it means to work communally. Through this sharing, we can see, touch, taste, and smell what our ancestors once ate, and in a way, whenever we eat Indigenous foods, we dine with our ancestors. The tears that flow from our program participants nourish the land and we honor their vulnerability by ensuring sovereignty is at the forefront of every program we produce.

For us this emotion that Indigenous foods bring out is a love that transcends time and space, connecting the past and present to the future. We see it in the smiles on Elders’ faces as they excitedly share recipes, in the laughter of children as they learn to sow seeds, in the glow of mothers as they share their sacred milk knowledge, and in the excitement of students as they take up the helm and share what they know. For far too long our knowledge has been taught in the past tense, but we are a living people. Our First Foods Program allows us to share in the abundance that this earth provides, and to remind ourselves that these gifts have always been ours. We have the sovereign right to reclaim our place as stewards of these lands and to decide how our knowledge is transmitted to the next generation.

## Kaitlyn Patterson, All Our Relations Land Trust, Katarokwi, Ontario, Canada

Boozhoo, Kaitlyn nindizhinikaaz miinwaa Wàbà Wàwàshkeshìkwe nindizhinikaaz. Maang nidoodem. Mattawang nindonjibaa. Elgin endaayaan. My name is Kaitlyn, my spirit name is white deer woman, and I am from the Loon Clan. I am a mixed ancestry Algonquin Anishinaabekwe and my Algonquin family is from Mattawa, Ontario (817 General List/Sudbury). I have lived most of my life in Katarokwi (the greater Kingston area) and am connected to the urban Indigenous community in Katarokwi as a result. I am a mother, aunt, sister, and partner. I am a registered dietitian as well as an Assistant Professor and Queen’s National Scholar in the School of Public Health Sciences at Queen’s University. I am passionate about IFS, particularly in urban settings, and how connections to lands and waters support Indigenous health and wellbeing.

In 2022, I cofounded All Our Relations Land Trust with fellow members of the Katarokwi urban Indigenous community. The land trust is an Indigenous-led charitable organization that aims to support and restore diverse ecosystems within Katarokwi and safeguard the life of the land for the benefit of past, present, and future generations of all our relations [12]. The term “all our relations,” refers to the concept that all living beings are connected to one another—we rely on each other to survive and flourish as interdependent members of Creation. And although this is true, I also acknowledge that, according to Anishinaabe Creation story, humans were the last beings created and are thus more dependent on our ecological kin (that is, plants, animals) than they are on us. This teaching emphasizes humility and affirms our roles and responsibilities within an ecosystem. With an all our relations worldview, the land trust engages in IFS activities that increase biodiversity and address the climate crisis locally. Through Indigenous-led action, our vision is to transform the way people conceptualize and relate with lands and waters to strengthen ecological kinships that protect the continuation of life.

All Our Relations Land Trust works on the traditional territories of the Anishinaabeg, Haudenosaunee, and Huron-Wendat, and within Dish With One Spoon Territory (which encompasses the Great Lakes region within Canada and the United States). Dish with One Spoon—also known as gdoo-naaganinaa (our dish)—teachings emphasize that humans should take only what they need, leave something in the dish for everybody else, and keep the dish clean [13,14]. As an urban Indigenous group working within the territory, we acknowledge and pick up our responsibility to care for the lands, waters, and more-than-human beings who support us within Katarokwi. Currently, the land trust cares for 2.86 acres of land on the east end of the city of Kingston. The land is owned by the United Church of Canada and was previously used as a hay field for nearly 3 decades. In 2020, members of the urban Indigenous community began caring for the space, and since that time, have started an IFS garden and planted over 1600 native trees and shrubs of ∼60 different species. In 2024, the land trust is preparing to accept this gift of land from the church, so that members of the urban Indigenous community can continue caring for the garden to fulfill gdoo-naaganinaa responsibilities in an area at risk of further urbanization within Katarokwi. At present, the east end faces pressures for more housing and industrial development as the city’s population grows, and caring for the garden is how we take direct action so that the beings who currently live in this area will survive.

At the garden, we grow a mix of conventional and Indigenous foods, and we priorities growing native species. The food we grow has been distributed throughout the community with help from local organizations including Kingston Native Centre and Language Nest, and Loving Spoonful. The medicines grown at the garden are saved for ceremonies occurring throughout the year and are shared among Indigenous community members who visit the space. *Semaa* (tobacco), *giizhik* (cedar), *maskodewashk* (sage)*, wiingashk* (sweet grass), and *ode’iminan* (strawberries) are some of the medicines you can find there. In this urban garden you can also find black walnut trees, *miskominag* (raspberries), serviceberries, black-eyed susans, milkweed, and oak, maple, pine, and pawpaw trees (to name a few)—all in this place located within city limits. The willow trees are growing well in the wet soil by the property line and in the spring you will hear the peepers, watch a *wabooz* (rabbit) dart by, and maybe even spot a *waawaashkeshi* (deer) on the hill overlooking the garden. Growing food in the city defies all assumptions and assertions that Indigenous People and our food systems do not exist here—that we do not belong here. On the contrary, the life we observe on the land, and the expanding biodiversity that has occurred here demonstrate that we must be here and that our work—the relationships—must continue.

A significant aspect of the land trust’s mission also involves community-based education and involvement. At the garden, we host visitors who want to learn more about the land and our IFS practices. In the past few years, the garden has welcomed hundreds of elementary, high school, and postsecondary students who have come to participate in ceremony and contribute to the space. Many of the trees and shrubs planted at the garden have been by children and youth who will watch the forests grow over time. Families with babies and young children also visit the garden and care for the land by planting, harvesting, singing, and learning their Ancestral languages with help from Knowledge Holders. This intergenerational learning and interaction demonstrate the role that everyone plays—from babies to elders—in caring for gdoo-naaganinaa. In our dish, every being has a responsibility including the animals who scatter seeds across the field, living organisms in the soil who support newly planted trees, and the water that flows from Butternut Creek located further east of the garden, offering the foundation of life. In our IFS practices, we honor these interdependent roles as we grow food that feeds people and strengthens food security for all our relatives who pass through and call this place home.

There is a long road ahead as we continue caring for the lands and waters in this urban place, yet we have made big impacts since the inception of the land trust only a few years ago. Ongoing advocacy and education, along with relationship building among community members and with Indigenous groups with similar goals, will strengthen our mission and impact. Although it is common for Indigenous presence and relationality in urban places to be ignored and denied, the work of All Our Relations Land Trust directly challenges these ideas as we foster connectivity, strengthen Indigenous food systems, and enhance biodiversity within Katarokwi.

## Dave Skene (Wisahkotewinowak Collective, Kitchener, Ontario, Canada)

“The streets are land too. For us who are displaced from our home communities, the inner city is our land-that’s our First Nation-that’s our home community…I was told we are the land. If the land raises you up, you are that land. What land raised me up? The North End of Winnipeg…. First Nations, Métis and Inuit people that are now urban dwellers. They are now the roses growing through the concrete” [15]. I wanted to open with this quote from Michael Redhead Champagne, a Cree youth worker from Winnipeg, Manitoba. I believe it paints a picture of who we are as Indigenous Peoples having made our homes in the cities of Turtle Island. I am Métis and for most of my life I have lived in cities, but it is only recently that I have begun to understand that the city can be a place where I find relationship with Land. Before 2016, I saw Land as something that existed outside of the city and would like to share a little piece of the journey I have been on since then to understand how the city can truly be a place where we can practice an Indigenous Land-based life.

Our identities as Métis people can be complex as our ancestry of different European and First Nations cultures as well as geographical diversities that are fundamental in defining who we are does not allow for a homogeneous Métis people. Although the Métis emerged from relationships between First Nation women and European men, it is important to note that we are not just a hybrid of 2 races. We are a whole people who come from a line of whole people. In my case the parts of Scottish, Cree, French, and Menominee came together through history to make a whole person—the person I am today. To further situate myself in this story, I also see myself as an urban Métis person. For most of my life the city has been the place I have called my home. As a Métis person I exist in relation to place. This is why I believe it is significant to state that I am an urban person. Living in the city is where I live out my relationship to place. Place is different from space as there is a greater emotional and ontological connection to place. This connection to place often leads to a sense of belonging and in many cases belonging and place can be used interchangeably. For some the city might seem an unlikely place for Indigenous People to find place, as cities are often seen as the ultimate expression of colonization and settler capitalism. The reality is that most cities are built on lands that have historically been gathering places for Indigenous Peoples. The issue is that those who most often hold the power of the city are part of the settler colonial systems and therefore creating cities in their own image. As a Métis person it is part of my responsibility to resist the colonial systems and find ways to disrupt the focus of settler activity. One of the ways I am involved with doing this is through the collaborative creation of urban place-based cultural practices that include our Indigenous relationship to the Land within the city. In doing our hope is to ensure that the life of the city includes the wellbeing of all Indigenous Peoples by providing the conditions for a flourishing community.

My connection to the city has raised a fundamental question: how do we as Indigenous Peoples living in the city still understand the roots of our identity as coming from the Land? I first started asking myself this question as I attempted to understand my own urban Métis identity. Land ties Indigenous Peoples to our systems of knowledge. It is the Land that informs our ethical engagement with the world and all our relationships with both human and nonhuman others. Our Indigenous knowledges do not disappear when we come to live in cities. So, I began to explore how to reflect an urban and local Indigenous understanding of how to engage with the Land. The idea of planting a garden seemed the simplest expression to explore this idea. In 2016, I hired 2 Indigenous students to work with me and start a garden. They also were asking a similar question around identity and Land. During that summer Alyssa, one of the summer students, and I took part in an Indigenous photo story project in Toronto. Alyssa was telling her story, and she made a statement about how she felt about herself as an Indigenous person: “I am saddened by how sometimes I get treated because of how white I look to the Native community and how Native I look to the white community.” Alyssa was expressing how she felt she did not really belong and I began to understand there were others who felt a little lost in who we are as. As we worked through the summer and I met other Indigenous youth, I heard similar stories in the garden. The 3 of us did not have the answers, but we knew that planting a garden together was pointing us in the right direction.

Upon reflection I see now that the garden was teaching us about place and belonging. The Land we were gardening on was at first a more abstract space. As we worked and built relationships with each other, the Land itself became more of a tangible place. Indigenous educator Sandra D. Styres understands that through relationship Land becomes place, which is, “conceptual, experiential, relational, spiritual, emotive and cognitive” [16; p.49]. Speaking of Indigenous identity as it relates to youth and the significance of Land, Styres goes on to state: “we need to find new ways to love the Land in essence we need to find different ways of (re)membering and (re)cognizing identity and meaning making from contemporary rural, urban, and mixed-blood landscapes.” As Indigenous People we have the ability to continually reconstruct and modify our worldviews, so new traditions can evolve to meet contemporary realities [16]. I believe this is what was happening in the garden that summer. We were beginning to imagine new ways to care for the Land and be cared for by the Land in the city. Although our focus was on revitalizing Indigenous Land-based practices, our goal was to continue to create places with the Land where Indigenous People in the city feel they belong.

I am not sure where I heard this statement, but it has stuck with me for a long time now—searching for the truth always involves searching for community. It did not take long for us to find others who were asking similar questions. Within a year there was a small group of us meeting regularly including Indigenous university professors, students and researchers, non-Indigenous friends, and Indigenous youth. To tie us all together we decided to call ourselves Wisahkotewinowak, an Indigenous Garden Collective. Wisahkotewinowak is a Cree/Michif word that means: the growth of new green shoots that come up from Mother Earth after a fire has come through the Land [17]. As a Collective or what could also be called an intentional community, we exist in relation with each other, with our wider community, and our nonhuman kin (the Land, plants, animals, and spirit) in the urban spaces we call home [18]. With these influences I began to understand that the youth I was being introduced to were reaching out for community, a place of belonging that included the Land as a significant aspect of their community.

As a Collective we began to understand that food is a conduit to understanding our place on the Land. Caring for seeds, planting them, supporting their growth, harvesting, preserving, and of course eating the food produced from the Land nourished us in body, mind, and spirit. We are also aware of the practical food security needs in our local Indigenous community, which led to us seek out places within the city where we could grow food to share. The Three Sisters Garden is located at Steckle Heritage Farm in Kitchener, which was once a large Mennonite farm. The farm is now surrounded by the city and has become a 10-acre educational farm. This is where we primarily grow tobacco, sunflowers, and the 3 sisters: corn, beans, and squash on ∼2 acres. Another garden is located at the University of Waterloo Environmental Reserve. We referred to this as the Produce Garden to reflect our focus on growing vegetables and fruit for cooking and preserving workshops. Food from this garden is harvested weekly and contributes to our Food Share program, which we provide in collaboration with White Owl Native Ancestry Association (WONAA). Not that you should have favorite gardens, but this is mine. It is a peaceful place and typically has a cool westerly breeze. The space is a host to hundreds of birds including Great Horned Owls, Red Tailed Hawks, Bald Eagles, and Ospreys. There is also a family of deer sharing the space and the food we grow. This Land is full of life.

The Land that speaks to me the most and is my favorite place to be is the White Owl Sugar Bush that is home to hundreds of maple trees. WONAA acquired the sugar bush through a land transfer from the United Church of Canada in 2016. The 10.5-acre mixed forest also contains oak, beech, and pine trees. Since the first boil in 2018, Wisahkotewinowak’s maple syrup production has steadily grown with an average of 130 L (34 gallons) a year. The syrup is shared with the Indigenous community through the Food Share program and seasonal events. The maple syrup season is a short and intense season, lasting only 7 wk. We set up our sugar camp toward the end of February when the temperatures are still below freezing, and the snow is still on the ground. Once the camp is established, we start taping the maple trees. Once this is done we let the Land and the weather do their jobs so we can collect the sweet water and boil it down to delicious syrup. As we work the Land around us begins to change and the new life that comes with spring begins to reveal itself. This is what I love about working in the sugar bush, watching the season change little by little each day. The Métis and First Nations People who lived in territories where the Sugar Maple are plentiful call this time the sugar moon and the process of harvesting the sap to make sugar was a celebration of renewal in our relationship to the Land.

## Christine and Clifton Bruno (Indigenous Consultants, Portland, Oregon, United States)

I, Clifton Bruno, am a Wasco Tribal member of the Confederated Tribes of Warm Springs. My name is Christine Bruno and my heritage is Basque, Comanche, and Irish. Together we have worked with schools, youth, and family programs for over 25 years sharing Indigenous culture, art, history, ethnobotany, games, first foods, and lifeways. We provide Indigenous Traditional Ecological Cultural Knowledge (ITECK) for many environmental and educational projects in the Portland Metro Area through professional consultation and on-site education. Our work includes collaborations with Metro (a regional government), Portland Parks and Recreation (PP&R) at the Native Gathering Garden (NGG) at Thomas Cully Park, Confluence Project, Portland State University, United States Forest Service at the Sandy River Delta, Portland All Nations Canoe Family and the Northwest Indigenous Food Sovereignty Alliance, NARA Youth Programs, and NAYA Youth and Family Center.

By building relationships with land-holding governmental agencies, from local to national, urban Indigenous Peoples access and spend time on these lands to tend, learn, harvest, and heal. ITECK is being respected by more of these agencies and Indigenous Knowledge Keepers are consulting on the restoration and management of these lands in and near Portland, Oregon. The urban Indigenous community of Portland Oregon is the ninth largest in the United States, representing all 9 federally recognized Tribes of Oregon and over 380 other Tribes [19]. In 2010, the Native American Community Advisory Council of PP&R brought together Indigenous individuals who recognized a need to improve representation, equity, and access regarding the 11,672 acres of parks, natural areas, and their governing bodies. Other land-holding agencies joined, including Metro, a 3-county regional government with over 18,000 acres of parks, natural areas, and land restoration projects. PP&R and Metro both have Indigenous Community Liaisons. Indigenous-led nonprofits, small businesses, and individuals are being contracted to do the ITECK work at many sites.

There are many ways we connect to our plant relatives growing in the diverse ecosystems across our Indigenous Lands. The urban areas are not formally part of a Reservation or Reserve but are still Indigenous Lands. Residing in the urban setting, we find ways to carry and intergenerationally share the traditions, lifeways, and foods that our people have held up while supporting the living beings and environmental systems around us.

In the Pacific Northwest of North America, we experience Seasonal Rounds that are following the natural changes of the sun, moon, tides, and weather patterns. These influence the harvesting and tending of the environment for plants and animals. At the Quamash Prairie, spring is a busy time and often starts before the Gregorian calendar marks the occasion. First fresh greens and blossoms are added to the diet along with roots and bulbs. The various species of Camas Asparagaceae, an herbaceous perennial found in riparian, meadow, and prairie environments, bloom from mid to late spring. Out at Quamash Prairie, the water recedes, and soil temperatures rise, allowing our plant relatives’ leaves to shoot up, changing the brown mud to green, followed by the brilliant blue and violet hues of the Camas flowers. We gather for ceremonies and celebrations of this ancient food. Knowledge sharing and connection to the natural world provide healing for Urban Indigenous Peoples. The birds’ dawn chorus awakes the morning fire tender, and the night crew retire to their tents. Prairie mist, and sometimes a bit of frost, catches the sun’s golden rays streaming over the mountain ridges. The deer graze nearby, nodding a greeting, their large curious eyes watching as the passerines and waterfowl are getting their morning meals all around us. Eagles, Hawks, and Herons glide above. It is not a sacrifice to take on this responsibility. It is a privilege that brings joy and healing with the community sharing the bounty.

In 2007 we toured this site that was named after the homesteader family that had occupied the land for the last 100 years. Even though there had been a working farm, the original creek, pond, and acres of ancestral Camas and Oregon White Oak habitat were intact. These farmers welcomed Tribal People to continue annual harvesting for many years, but eventually demands of modern times stopped them from coming, but this was soon to change with new generations of Indigenous Peoples interested in our first foods. Traditional digging sticks and modern tools break into the soil and reveal the nutrient-packed, starchy bulbs. Continued restoration and tending occur by removing invasive species and dense clusters of Camas bulbs are redistributed to other areas of the 160-acre natural area. Without this careful hand harvesting and tending, the Camas will become too crowded, and bulb size will decrease. All ages work and learn together. Many of the adults will celebrate their first time connecting with Camas or restarting a family tradition. These young children will always know this plant relative and look forward each year to building their relationship and taking their part in the reciprocity of caring for this environment. The community gathers to on the final day to experience multiday pit cooking techniques that will turn the white bulbs into a sweet, amber-colored delight. Spring Chinook Salmon are also prepared traditionally and cooked over the fire to make up the shared feast.

At the NGG, the springtime sun rises over Mount Hood, warming an Anna’s Hummingbird perched on a blossoming Chokecherry tree. This Hummingbird’s territory is filled with many other animals and he will announce this presence to school children learning about native plants and a group of urban Indigenous People tending the land. This Hummingbird’s ancestors remember the free-flowing waters of Ni’chi Wana River that flowed through the land rich with plant and animal relatives whose coexistence supported large villages each with 1000–5000 or more people. Through processes of colonization, the Indigenous Peoples were forcibly relocated, confined to reservations, given European names, and forbidden to follow their usual seasonal rounds to obtain first foods. Likewise, Ni’chi Wana was renamed Columbia River, and their waters were moved away from the wetlands and floodplains. These lands became occupied by homesteading and farming, which later gave way to industrial uses in the growing cities of Portland, Oregon, and Vancouver, Washington. In 1936, a sand and rock quarry, in the Cully neighborhood of Northeast Portland, provided building materials and when that resource was spent in 1978 the large pit became a construction waste dump. In 1990, the garbage-filled pit was closed, topped with a liner, and covered with soil. From 2012 to 2013, several PP&R design planning sessions with the Native American Community focused on a quarter-acre section of the 25-acre Thomas Cully Park. In the meetings, Native American Community members expressed desired uses for the space, including a place for ceremonies and events, native plantings, and culturally significant architectural design elements. This section would grow to the existing 8-acre NGG, which opened in 2018. There was disappointment in the land offered for the NGG being a former landfill, but a Tribal Elder spoke up, saying, “who better to heal the land, than us.” This statement has led to the motto, “as we heal the land; we heal ourselves.” Today the NGG houses numerous native plants including tiny mosses, flowers, shrubs, and smaller trees, planted guilds with irrigation, nonirrigated areas, and a grassland prairie. Basalt stones provide seating and are placed to mark the 4 directions and the surrounding mountains.

## Sara Moncada (The Cultural Conservancy, San Francisco, California, United States)

Warm greetings. My name is Sara Moncada. I carry Yaqui ancestry from my father and predominantly Irish ancestry from my mother. It is important for me to acknowledge the songs that sing in my blood through my mother’s side as deeply as I acknowledge those songs that sing in me from my father that connect me to this beautiful continent. Through her I carry a tie to a First People’s relationship to the lands and waters of a place oceans away, and those deep knowledge systems and ways of knowing and being. In many ways, this connection strengthens my responsibility. I am connected to a long story of land relationships across the earth and that those places and traditional knowledge systems meet within me. I am honored to share a bit about the good work of The Cultural Conservancy (TCC) and our commitment to revitalizing Indigenous cultures, lands, and community wellness. We serve as both a local and global organization and since 2019, as a land-based organization. We share our work through community programs and Indigenous media, including The Native Seed Pod podcast series that explores and celebrates traditional seeds, Native Foodways, and TEK.

TCC is based in San Francisco, California, on unceded Ohlone land. Since our founding in 1985, our mission is to protect and revitalize Indigenous cultures through the direct application of traditional knowledge and practices on ancestral lands. For almost 40 years, we have worked across Turtle Island and Moana, with California Indian communities, American Indian Nations, Kānaka Maoli, and other Pacific Islanders, and Indigenous communities in Central and South America. We work on a wide variety of community-based projects, from sacred site protection to the conservation of endangered languages, the preservation of song and arts traditions, and the revitalization of Native Foodways. Critical to our work is the acknowledgment of the sacred relationship that Native peoples have to their lands and waters and the importance of this relationship to their physical, mental, and spiritual health.

Our regional work efforts focus on serving the urban Intertribal and California Native communities of the greater San Francisco Bay Area including hundreds of Tribal Nations and dozens of local California Indian tribes. Because of federal relocation policies that forced Native people off reservations, genocidal practices that decimated California Native communities, and continued economic, political, and cultural dispossession, these displaced urban communities face many unique challenges. Without access to land and ancestral territories on which to gather, grow, and practice land-based traditions, they have experienced devastating losses of cultural traditions and intergenerational ecological knowledge of ancestral plants and seeds. By creating opportunities and increasing access for Native community, traditional land tenders, gatherers, and land stewards, we facilitate community-led transformation for those struggling with food access, land displacement, and community wellness.

We collaborate in educational programming, food and seed distribution, and cultural resilience. Our community work is strengthened by the diversity and creativity of local Native partners, in part a reflection of revitalized traditional trade routes and allyships. Those who we collaborate with locally include: Sogorea Te’ Land Trust, California Indian Museum and Cultural Center, Native American Health Center (San Francisco and Oakland), American Indian Child Resource Center, Wahpepah’s Kitchen, and Sonoma County Indian Health Project’s Elders and Families program. We are also honored to be on the Advisory Council for the new American Indian Cultural District of San Francisco, the first of its kind in the Nation. We continue to deepen our connections, collaborations, and learning exchanges with national and international Native food sovereignty groups such as Indigenous Seed Keepers Network, Native American Food Sovereignty Alliance, Intertribal Agriculture Council, and Slow Food Turtle Island Association that we helped to co-found.

Central to the implementation of TCC’s organizational mission is our Native Foodways Program. This keystone program is centered on the revitalization of traditional foods, seeds, and culturally relevant land-based practice, and supports land access and the food sovereignty of the Bay Area Native community region. In addition, our Native Foodways Program work contributes to the larger IFS and food justice movements regionally, nationally, and globally. Our community food distribution work through the Native Foodways Program is a deep reflection of our commitment to community service and land-based self-determination. Through this program we have distributed local, organic produce and Native-grown traditional foods, seeds, traditional recipes, and traditional foodways learning opportunities to Native community partners for over a decade to health centers, schools, and youth groups. Since 2019, through our land project Heron Shadow, we steadily increased our food distribution in response to continued heightened community need since the pandemic, providing increased poundage each year of fresh, nutritious, and culturally appropriate food throughout the intertribal Bay Area community. Building on the foundations of this good work, we continue to strengthen and grow our Native Foodways Program to bolster the food sovereignty, health, and self-sufficiency of our local community.

In 2019, we became a land-based organization. After 35 y of growing our organization and implementing our mission to protect and revitalize the sacred relationship Native peoples have with ancestral lands, we become stewards of our own land—a 7.6-acre parcel in Sonoma County, on the ancestral territories of the Coast Miwok and Southern Pomo peoples. This arable plot of land is becoming Heron Shadow: an Indigenous Biocultural Heritage Oasis, a community home for the dreaming, building, growing, and transmission of TCC’s mission and work. We are transforming this land into an innovative haven that focuses on the protection and regeneration of Indigenous agriculture, Native sciences, and healthy lifeways. At Heron Shadow we work to create a safe space for intergenerational learning and exchange that celebrates the complex systems of Indigenous knowledge and practices. It is a place of learning for Indigenous communities to engage in Native ecological practices, such as traditional fire tending, through the lens of California Native peoples and TEK systems. It is a priority to bring these diverse knowledge systems and learning models to the forefront of addressing ecological and climate problems so that we can heal our Mother Earth. Through our on-site programs, we plant seeds with the learners who visit and in turn support their capacity to grow, cultivate, and teach these lessons in their own communities in culturally appropriate ways.

Through Heron Shadow, our long-term goal is to develop a mission-driven educational space that serves our Native communities, Native farmers and food producers, Native land tenders and permaculturists, and traditional practitioners of the Bay Area and beyond. With our increased capacity through this land, Heron Shadow has allowed us to grow our local food justice program exponentially. Before this gift of land, we were honored to grow, source, and distribute 500–1000 pounds of heirloom foods and seeds to the community annually. With the addition of the Heron Shadow land base, in 2023 we grew, sourced, donated, and distributed 16,000+ pounds of nutritious, culturally appropriate foods to Native communities including plant starts and Native heirloom seed packets supporting community gardens, family plots, and land revitalization projects. Our goal is to thoughtfully continue to grow these numbers while we ensure our work remains sustainable, collaborative, far-reaching and deeply impactful with the communities we serve. Our Foodways Program would not be possible without all TCC board, staff, community leaders, and supporters who have contributed to this work over the decades.

We work closely with our Native Advisory Council, community partners, and networks to vision, plan, and implement programs that are foundationally rooted in and guided by community visions, requests, feedback, and guidance. We recognize that every Indigenous community has its unique definition and plan for self-determination, equity, justice, and kinship. Our community support is defined by collaboratively creating with our partners and deeply listening to the teachings and guidance of Elders, youth, and the more-than-human kin who we are in reciprocal relation with.

## Working in Relationship

These reflections and knowledges shared in the form of stories as part of the panel and further embodied by the relationships that continue to be formed within the North American Urban Indigenous Food Systems network illustrate the evolution of Indigenous foods and Indigenous Peoples across Turtle Island. Through the 5 narratives, 7 authors shared who they are as Indigenous People living in the city as well as the pride and meaning in what they do. Challenges and the unique circumstances faced and overcome were also conveyed along with the structural barriers associated with diverse urban environments. Several of their stories also included illustrative descriptions of the lands, waters, plants, animals, and humans encountered in their work. As expressed by Alex and Brooke, it is imperative to share these experiences, values, and histories to build connections across those spaces and places that are unique to Indigenous Peoples living in urban contexts. As Dave reflected on, cities can be places for Indigenous communities to thrive because connection to place and finding relationship with Land leads to a sense of belonging that can be elusive.

The sense of belonging and connection along with experiencing the Land and all its gifts was mentioned by all the authors. Sara talked about the sacred relationships that Indigenous Peoples have to their lands and waters and how these are linked to health and wellbeing. Christine, Clifton, Kaitlyn, and Dave vividly described the beauty of the Land and all its gifts that provide a sense of healing. These “safe spaces,” as referred to by Sara, thereby create opportunities for intergenerational learning and exchange of traditions, lifeways, and foods. Through much of their work and programming, knowledge sharing, and connections to the natural world provide healing through relationship for urban Indigenous People.

By sharing IFS initiatives and practices through our growing urban network, that connection to Land and culture thereby supports and nurtures community. As Brooke and Alex shared, those spiritual and emotional connections associated with Indigenous foods encourage us to “dine with our ancestors” and that food is more than nourishment. There is an emotional connection that food provides beyond spaces and places that unites Indigenous Peoples across Turtle Island. Kaitlyn, Christine, and Clifton also stressed those ecological kinships and responsibilities that are uniquely held in urban locations to challenge and defy assumptions that Indigenous food systems and Indigenous Peoples do not exist in the city [6,18].

Higher food insecurity rates in urban areas have been attributed to limited access to foods obtained through subsistence activities, exclusion from reservation or reserve-based programs, and separation from community or family supports [20]. Yet the work being done by the groups profiled addresses these trends and complex issues through supportive, sustainable, and restorative practices of food system cultivation for urban Indigenous communities [3]. The incorporation of Indigenous knowledges and food practices within urban food systems and diets is a critical exercise of agency [7], to foster intergenerational and cross-community knowledge transfer [3]. The work these individuals and organizations are leading embody these global recommendations with the urban initiatives profiled in essence, building community around food.

IFS, as discussed throughout these narratives, is recognized as a framework supportive of Indigenous health, wellbeing, and environmental sustainability [21]. It is a diverse set of pathways taken up by Indigenous Peoples who actively maintain and continually strengthen their relationships and ecological responsibilities with land and food [22]. IFS recognizes food as sacred, while underlying the active participation and decision-making from Indigenous Peoples that is necessary within food systems while highlighting the need for supportive policies and legislation to strengthen Indigenous lifeways [23]. Through an IFS approach, Indigenous Peoples are restoring food environments that host a balance of Indigenous foods and practices, while building strong community supports and culturally safe spaces where Indigenous communities can gather to heal and flourish in cities despite the enduring impacts and encroachments of settler colonialism [24,25].

As Brooke and Alex expressed, there is sovereignty in reclaiming place and belonging as caretakers of these lands and deciding on how new knowledges are transmitted to the next generations. Cities across Turtle Island are still Indigenous Lands as Christine and Clifton continue to model within their community. There are many ways to connect with plant relatives growing in these diverse ecosystems to heal the Land and heal ourselves. As an emerging and evolving network, there is hope for the shared responsibilities, new connections, and relationships that lie ahead.

## Author contributions

The authors’ responsibilities were as follows – HN: prepared the introductory and conclusion sections; Christine Bruno, Clifton Bruno, AC, SM, KP, BR and DS wrote their individual sections of the paper; and all authors read and approved the final manuscript.

## Funding

This research was funded by the Canada Research Chairs Program.

## Conflict of interest

The authors report no conflicts of interest.
